# The Impact of Reactive Ionic Liquids Addition on the Physicochemical and Sorption Properties of Poly(Vinyl Alcohol)-Based Films

**DOI:** 10.3390/polym12091958

**Published:** 2020-08-29

**Authors:** Guoqiang Li, Edyta Rynkowska, Kateryna Fatyeyeva, Joanna Kujawa, Krzysztof Dzieszkowski, Andrzej Wolan, Stephane Marais, Corinne Chappey, Zbigniew Rafiński, Wojciech Kujawski

**Affiliations:** 1 Nicolaus Copernicus University in Toruń, Faculty of Chemistry, 7, Gagarina Street, 87-100 Toruń, Poland; grantli@doktorant.umk.pl (G.L.); edyta.rynkowska@wp.pl (E.R.); joanna.kujawa@umk.pl (J.K.); dzieszko@doktorant.umk.pl (K.D.); wolan@chem.umk.pl (A.W.); payudo@chem.umk.pl (Z.R.); 2 Normandie Univ, UNIROUEN, INSA Rouen, CNRS, Polymères, Biopolymères, Surfaces (PBS), 76000 Rouen, France; stephane.marais@univ-rouen.fr (S.M.); Corinne.Chappey@univ-rouen.fr (C.C.)

**Keywords:** poly(vinyl alcohol), reactive ionic liquids, polymer membranes, sorption kinetics

## Abstract

A new type of hybrid polymeric-based film containing 1-(1,3-diethoxy-1,3-dioxopropan-2-ylo)-3-methylimidazolium bromide (RIL1_Br) and 1-(2-etoxy-2-oxoethyl)-3-methylimidazolium bromide (RIL2_Br) reactive ionic liquids was elaborated. Poly(vinyl alcohol) (PVA)-based films with 9–33 wt % of RILs were subsequently characterized using Fourier transform infrared spectroscopy with attenuated total reflectance (FTIR-ATR), scanning electron microscopy (SEM), atomic force microscopy (AFM), thermogravimetric analysis (TGA) and TGA-FTIR. PVA-RIL films were also studied in tensile tests, contact angle and sorption measurements. RIL incorporation enhanced thermal and mechanical stability of PVA membranes due to the hydrogen bonds between RILs and polymer chains. Membrane swelling behavior in water (H_2_O), ethanol (EtOH), and propan-2-ol (IPA) and the kinetics of water sorption process revealed that PVA-RILs membranes possess the highest affinity towards water. It was pointed out that both the RIL type and the RIL amount in the polymer matrix have significant influence on the membrane swelling behavior and the water sorption kinetics.

## 1. Introduction

Membrane-based technology has been playing a significant role in pervaporation [1,2,3,4] and gas separation process [5,6,7,8,9] due to its numerous advantages. Polymeric membranes have been intensively studied and applied in many processes due to their good processability, low cost and reasonable long term performance [8]. Poly(vinyl alcohol) (PVA) is indicated as one of the most versatile polymers used for the elaboration of membranes selective towards CO_2_ (for gas separation) and hydrophilic compounds (for pervaporation). In general, PVA and PVA-based polymers possess high film-forming abilities, good chemical stability, and high mechanical strength that allow their wide application [9].

Polymer membranes usually show the trade-off between selectivity and permeability, resulting in their separation performances under the Robeson upper bound curve [10]. Many researchers have been working on the modification of PVA-based membranes for gas separation processes to improve simultaneously their selectivity and permeability, namely, to break the trade-off relationship of PVA membranes [7,8,11,12]. PVA polymers have been also widely studied in the dehydration using pervaporation process owing to the excellent PVA hydrophilicity resulting from the presence of the hydroxyl groups in the polymer structure. To develop PVA-based membranes with both high selectivity and high permeability, Zhang et al. [13] blended chitosan (CS) with PVA to form PVA-CS membranes. The swelling degree of PVA membrane in water decreased significantly when it was blended with CS. More importantly, the separation factor and total flux equal to 27,000 and 402 g·m^−2^·h^−1^, respectively, were achieved at 40 °C in *n*-butyl acetate/water (98/2 wt %) binary system when the blend membrane contained 25 wt % of CS [13]. The preparation of mixed matrix membranes (MMMs) is commonly used to enhance the membranes performance. Penkova et al. [14] elaborated MMMs by fullerenol C_60_(OH)_12_ incorporation into PVA-based membranes for pervaporation dehydration of alcohol. It was found that *n*-butanol sorption increased after the incorporation of fullerenol into the membranes due to the hydrophobic-hydrophobic interactions between hydrophobic aliphatic parts in butanol structure and hydrophobic fullerene ring in fullerenol structure as well as by formation of hydrogen bonds between –OH groups in butanol and fullerenol. However, all PVA membranes showed strong affinity for water. Membranes containing 5 wt % fullerenol and 35 wt % maleic acid possess separation factor equal to ca. 7 and the highest flux equal to 600 g·m^−2^·h^−1^ in the *n*-butanol/water (42.5/57.5 wt %) mixture separation process, among all the prepared membranes.

According to the aforementioned discussion, polymer-based membranes are generally modified to ameliorate their physicochemical properties and separation performance in various applications. A tremendous number of possibilities of modification on polymeric membranes, including polymer blending [15,16], crosslinking [12,17], surface modification [18] and the incorporation of additives into polymer matrix [7,19,20,21,22] have been studied. Another alternative approach to modify polymeric membranes is the addition of ionic liquids (ILs) into the polymeric membranes.

ILs are organic salts containing organic cations and organic or inorganic anions. They possess many distinguished physicochemical properties including inconsiderable volatility, non-flammability, good ionic conductivity [23], high thermal stability and chemical resistance, CO_2_-philicity as well as solubility in many organic solvents [24,25,26,27,28,29]. The reactive ionic liquids (RILs) possess all the properties of ILs. Moreover, they contain functional groups, which can react with polymers to create covalent bonds between RILs and polymers. For instance, (3-(1,3-diethoxy-1,3-dioxopropan-2-yl)-1-methyl-1H-imidazol-3-ium) RIL was used for the elaboration of dense cellulose acetate propionate (CAP) based membranes [30]. CAP modification with RIL via the transesterification reaction led to the partial substitution of CAP functional groups with RIL. That led to the change of mechanical properties, thermal properties and swelling ability [30]. Therefore, polymeric membranes with the ionic liquids have found various applications including pervaporation [1,2,3], gas separation [24,31], and proton exchange membrane fuel cells [32,33,34].

Zhang et al. [3] prepared an IL-functionalized composite membrane by grafting the acidic IL into the poly(vinyl alcohol) chain to enhance the ethanol-acetic acid esterification reaction in a pervaporation catalytic membrane reactor. It was found that the PVA and acidic IL linked by covalent bonds can enhance the immobilized ILs stability and provide significant catalytic activity. The acid conversion was equal to 93% during 12 h at 75 °C with 20% catalyst content that was equivalent to the 19% enhancement of acid conversion compared to an equilibrium conversion achieved in a batch reactor under the identical conditions [3]. Klepić et al. [9] prepared blend PVA-based membranes containing 1-ethyl-3-methylimidazolium dicyanamide ([EMIM] [DCA]) ionic liquid by solution casting method for effective CO_2_/H_2_ separation. It was found that the [EMIM] [DCA] addition of 53 wt % into PVA-based membranes led to the raise of CO_2_ and H_2_ permeability, the decrease of the structural polymer chains regularity in PVA structure, and the change of crystalline phase orientation. Compared with PVA membranes, the glass transition (*T*_g_) and the melting (*T*_m_) temperature of PVA-IL membranes decreased from 67 and 164 °C to −40 and 111 °C, respectively. Safna Hussan et al. [35] reported an approach to ameliorate PVA properties by using 1-ethyl-3-methylimidazolium thiocyanate [EMIM] [SCN]. The PVA-based membranes with 20 wt % of IL possessed *T*_g_ equal to 66 °C and revealed the improved performance and thermal stability, decreased crystallinity, and the highest ionic conductivity among tested membranes [35].

The addition of RILs into polymer membranes changes mechanical properties, thermal stability, structural properties and the performance of membranes for different applications owing to the interaction between RILs and polymer chains. Therefore, the aim of this work was to fabricate the PVA membranes incorporated with different types and concentrations of ILs and to evaluate the effects of ILs on the physicochemical properties of PVA membranes. PVA-based membranes with incorporated RILs were successfully fabricated by casting from the solution and their physicochemical properties were characterized by FTIR, SEM, AFM, TGA, differential thermal analysis (DTA), derivative thermogravimetry (DTG), contact angle, tensile tests and swelling measurements in the solvents of different polarities. The kinetics of water sorption into membranes was analyzed by applying kinetics models. This work provides a deep insight into the morphology, thermal stability, mechanical strength and swelling behavior of RILs incorporated PVA membranes.

## 2. Experimental

### 2.1. Materials

PVA powder (Elvanol 71-30, fully hydrolyzed, a molecular weight of 100 kDa) was kindly provided by DuPont, USA. Chloroform, diethyl ether, ethanol, ethyl acetate, methanol and propan-2-ol were purchased from Sigma-Aldrich (Poznań, Poland) or Avantor Performance Materials Poland S.A. (Gliwice, Poland). Reagents of the analytical grade and the ultrapure water from Millipore^®^ (Fontenay-sous-Bois, France) deionized by Milli-Q system (18.2 MΩ·cm) were used for the ionic liquids synthesis or swelling measurements.

### 2.2. Reactive Ionic Liquid (RIL) Synthesis

**Figure 1:**
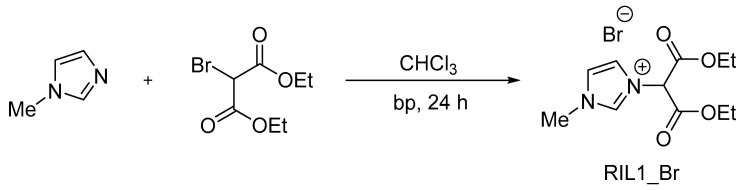
Scheme of 3-(1,3-diethoxy-1,3-dioxopropan-2-yl)-1-methyl-1H-imidazol-3-ium bromide (RIL1_Br) synthesis.

**Figure 2:**
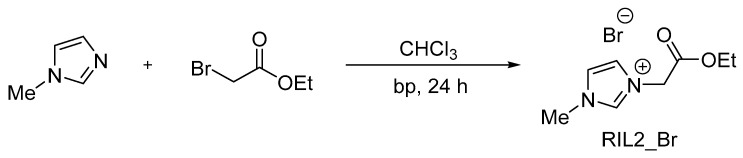
Scheme of 1-(2-ethoxy-2-oxoethyl)-3-methylimidazolium bromide (RIL2_Br) synthesis.

The synthesis was carried out according to the procedure described elsewhere [36]. The reaction yield was equal to 99% and 95% for RIL1_Br and RIL2_Br, respectively. The RIL1_Br and RIL2_Br structures were confirmed by ^1^H and ^13^C NMR analysis. The detailed NMR results can be found in our previous study [36]. The number of protons determined via integration of peaks in the ^1^H NMR spectrum, signals multiplicity and chemical shifts correspond to the proposed structure of RIL1_Br (Figure 1) and RIL2_Br (Figure 2). Moreover, all carbon atoms in a different chemical environment were matched to the signals in the ^13^C NMR spectrum [36].

FTIR-ATR ν_max_ (RIL1_Br): 3070, 2993, 1759, 1556, 1221, 1263, 1471, 1444, 1182, 839, 750 cm^−1^.

FTIR-ATR ν_max_ (RIL2_Br): 3425, 3082, 2982, 1745, 1572, 1225, 1470, 1439, 1174, 876, 766 cm^−1^.

### 2.3. Elaboration of PVA-RIL Films

**Figure 3:**
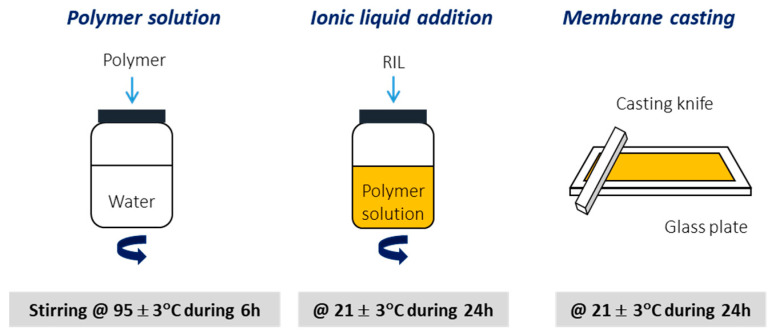
General scheme of poly(vinyl alcohol) (PVA)-RILs films fabrication.

PVA-RIL films were prepared by the addition of a given amount of RIL to the polymer solution, using water as a solvent. The obtained polymer-RIL solution was cast on a glass plate and subsequently membranes were formed using the phase-inversion technique induced by the evaporation of solvent. The prepared films were placed in an oven at 30 °C for 24 h to remove the residual solvent (Figure 3).

### 2.4. Membrane Characterization

Fourier transform infrared spectroscopy with attenuated total reflectance (FTIR-ATR) spectra were recorded using a Nicolet FT-IR apparatus (ThermoFischer, Avatar 360 Omnic Sampler) from 4000 to 500 cm^−1^ with a 4 cm^−1^ resolution and 256 scans using germanium crystal [37].

The top surface morphology of PVA-RIL films was observed at 30 keV using SEM microscope (Quanta 3D FEG). The cross-section images of PVA-RIL films were preceded by fracturing films samples in liquid nitrogen. The samples were sputtered with an Au/Pd conductive layer (80/20) with the 2–6 nm thickness. The detailed description of SEM measurements can be found elsewhere [36,37].

The PVA-RIL films surface topography was examined with an atomic force microscope (AFM) with a NanoScope MultiMode SPM System and NanoScope IIIa and Quadrex controller (Digital Instrument, Veeco, London, UK) and subsequently evaluated using root mean square roughness (*R_q_*) and arithmetic average height (*R_a_*) parameters. *R_q_* and *R_a_* parameters were determined for the 5 µm × 5 µm scanned sample area by using a Nanoscope v6.11 software (Bruker Corporation, Berlin, Germany) [36,38].

The hydrophobic/hydrophilic surface properties of the PVA-RIL films surface was determined from the total surface free energy (*SFE*) value based on the direct and indirect methods, namely by contact angle measurements (CAM) and an Owens-Wendt method [39,40]. CAM were conducted at 22 ± 3 °C and ca. 50% RH using water (Milli-Q), glycerol, and diiodomethane. The surface tension parameters of water, glycerol, and diiodomethane can be found elsewhere [37,39,41]. The measurements were performed utilizing the Multiscope apparatus (Optrel, Sinzing, Germany) with an accuracy of ±3° and applying the sessile drop method [37].

The thermal stability of PVA-RIL films was examined in the 25–800 °C temperature range, at 10 °C min^−1^ heating rate, and 90 mL min^−1^ nitrogen flow rate using the TGA thermogravimetric analyzer (TGA Q 500, TA Instruments, New Castle, DE, USA).

Thermal features were determined by applying Jupiter STA 449 F5 (Netzsch, Berlin, Germany) combined with FTIR spectrometer (Vertex 70 V Bruker Optik, Berlin, Germany). Thermogravimetric analysis (TGA), differential thermal analysis (DTA) as well as derivative thermogravimetry (DTG) were performed in an ambient environment of nitrogen at the range of temperature of 25–1100 °C. The heating rate was equal to 20 °C min^−1^. The thermal resistance and sample stability were defined according to the TGA and DTG. DTA provides the data related to the changes in the materials energy (enthalpy of the reaction and specific heat capacity). The application of TGA analysis coupled with FTIR gave the opportunity to analyze continuously the evolved gas products in 2 s intervals with the Netzsch-Proteus analytical system. FTIR collects 16 scans/0.3 °C with a resolution of 4 cm^−1^. The Gram–Schmidt Fourier transform provides the corresponding spectra for each time interval. Gram–Schmidt diagram was used for evaluation of the temperature range where the production of the gas is the highest. FTIR spectra were recorded in the range of wavenumbers from 4000 to 400 cm^−1^ with a resolution of 8 cm^−1^.

The mechanical properties of PVA-RIL films were examined for samples with 30 mm length and 5 mm width at 23 ± 2 °C and 43 ± 5% relative humidity using Instron 5543 machine (Instron, Norwood, MA, USA) applying crosshead speed of 1 mm·min^−1^ [37]. The mechanical strength of examined films was assessed taking into account Young’s modulus (*E*), elongation at break (*ε_max_*), and stress at break (*σ_max_*) parameters [42].

### 2.5. The Measurements of Sorption Processes in Water (H_2_O), Ethanol (EtOH), and Propan-2-ol (IPA)

All PVA membranes swelled very fast in the contact with water. To record the water sorption process accurately without any disruption, the sorption process was monitored under a microscope equipped with a camera (Microscope—MicroCapture Pro, Wels, Austria) at room temperature (25 °C) and atmospheric pressure. Firstly, the equipment was calibrated by using a calibration paper. Subsequently, a PVA-RIL film sample was placed in a glass Petri dish and the Petri dish was mounted under the microscope. A picture of the dry membrane was taken and stored on computer immediately. After that, a proper amount of distilled water was poured into the Petri dish and the picture of the membrane in distilled water was taken over a short period of time measured by stopwatch. The measurements were stopped when the size of the membrane became constant. All the diameters of membranes were measured and the area of each membrane was calculated. The ethanol sorption process and IPA sorption process were monitored according to the same procedure. The swelling degree (DS_t_) was evaluated according to Equation (1):

(1) $${\mathrm{DS}}_{t}{\;=\;(S}_{t}\;-\;S_{d})/S_{d}\;\times \;100\%$$

where S_d_ is the area of dry film, S_t_ is the area of the wet film during the swelling time t.

The swelling degree at equilibrium (DS_e_) was calculated by using Equation (2):

(2) $${\mathrm{DS}}_{e}{\;=\;(S}_{e}\;-\;S_{d})/S_{d}\;\times \;100\%$$

where S_d_ is the area of dry film, S_e_ is the area of the wet film at equilibrium.

## 3. Results and Discussion

### 3.1. Physicochemical Properties of PVA-RIL Films

#### 3.1.1. RILs and PVA Characterization

**Figure 4:**
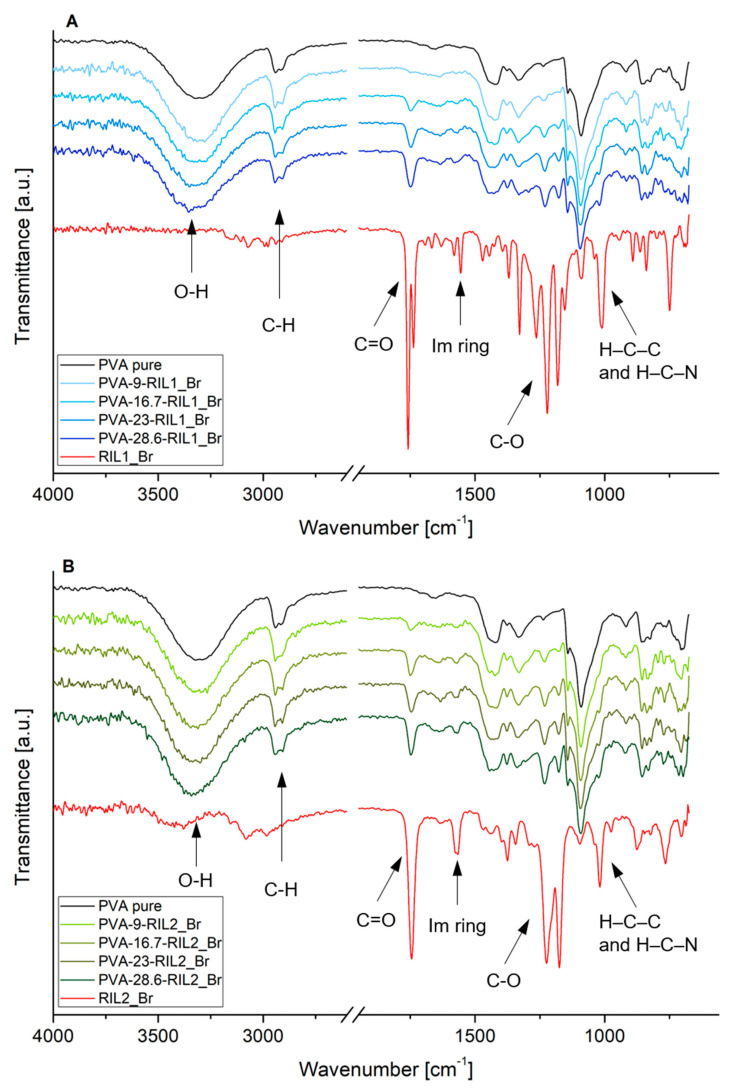
FTIR-ATR spectra of pure PVA, pure RILs and PVA-based RIL films with (**A**) RIL1_Br and (**B**) RIL2_Br at various ionic liquid content.

The presence of interactions between RIL and PVA was determined using FTIR-ATR analysis. The obtained spectra of pristine PVA film, pure RILs (RIL1_Br and RIL2_Br), and PVA-RIL films were normalized according to the –CH_2_– stretching vibration band (ν = 2939 cm^−1^) in the PVA structure [43]. The results are depicted in Figure 4. The performed FTIR-ATR analysis indicated the imidazolium (Im) ring stretching vibration bands at 1556 and 1572 cm^−1^ and bending vibration bands at 1182 and 1174 cm^−1^ (Table 1) in the case of pure RIL1_Br and RIL2_Br [44].

The FTIR spectra of PVA-RIL1_Br and PVA-RIL2_Br films revealed the intensity change of vibration bands due to the presence of IL as the membrane component. It can be seen that the increase of RIL content results in the intensity raise of C=O and C–O stretching bands in –COO– ester groups. This results from the higher concentration of functional groups responsible for individual vibrations. The blending of RIL1_Br and RIL2_Br with PVA intensifies also the stretching band of –OH groups in the PVA backbone. This band shifts to higher frequency values (from 3313 cm^−1^ for pure PVA to 3344 and 3334 cm^−1^ for PVA-28.6-RIL1_Br and PVA-28.6-RIL2_Br, respectively). Such behavior was linked to the strong interaction between PVA hydroxyl groups and RILs imidazolium-based cations. The presence of numerous –OH groups in PVA structure induces strong intramolecular hydrogen bond interactions between PVA and RILs [34,45]. The hydrophobic ethyl substituents attached to the hydrophilic ester-functionalized imidazolium ring in used RILs determine their amphiphilic nature. The RILs functionalization with ester groups also strengthens the hydrogen bond interactions between PVA and RIL [46,47]. As a result, the O–H bond in PVA becomes more durable shifting to higher vibrational band frequencies [45]. The similar phenomena was also noted by Rozik and Ward [48] and Patachia et al. [49].

The addition of RILs resulted also in the shift from 825 to 835 cm^−1^ of the skeletal and C–O stretching band for tested PVA-based membranes that was correlated to the increased PVA chain rigidity as a result of the strong hydrogen bonds between –OH groups [49]. Additionally, the ionic interactions between bromide anion and imidazolium cation lead to the changed H–C–C and H–C–N bending vibration in imidazolium ring from 1182 cm^−1^ (for pure RIL1_Br) and 1172 cm^−1^ (for pure RIL2_Br) to 1170 cm^−1^ (for PVA-RIL membranes).

Patachia et al. [49] pointed out concentration dependence of 1-butyl-3-methylimidazolium tetrafluoroborate ionic liquid [BMIM][BF4] on FTIR spectra of PVA/[BMIM][BF4] gels. The addition of [BMIM][BF4] to PVA-based gels resulted in the shift of the C–O stretching vibrational band to 844 cm^−1^ in respect to native PVA (830 cm^−1^) due to the PVA stiffening after the improved hydrogen bonds interactions.

#### 3.1.2. Thermal Properties

**Figure 5:**
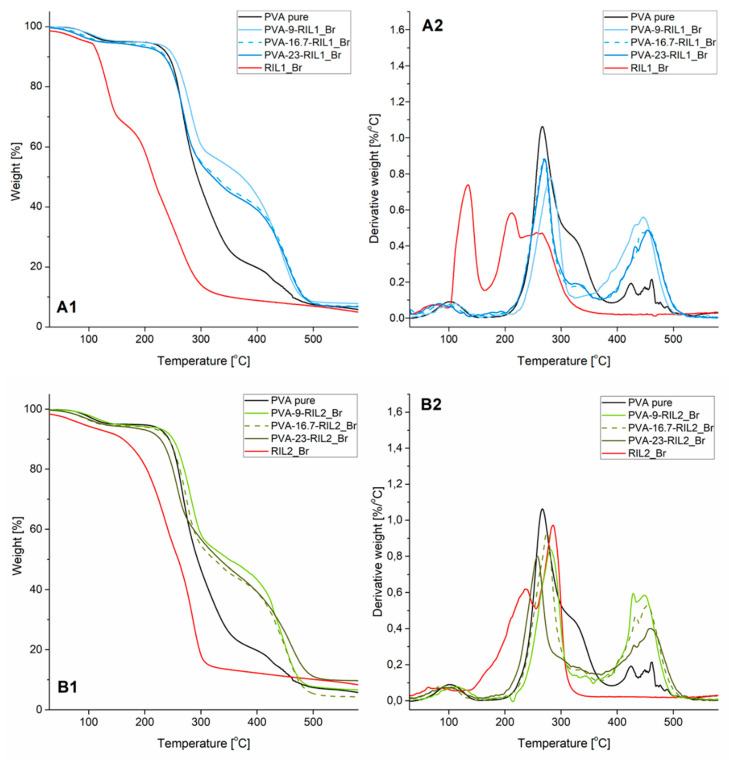
Thermogravimetric (TG) (**A1**,**B1**) and derivative thermogravimetry (DTG) (**A2**,**B2**) analysis of PVA-RIL films.

**Figure 6:**
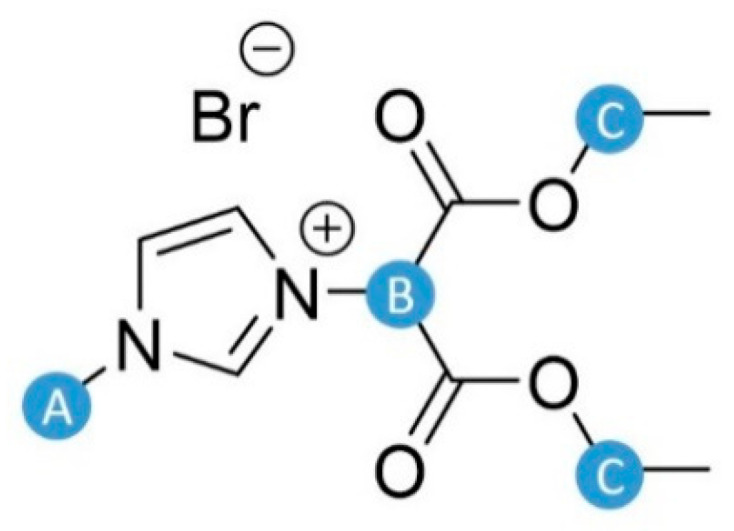
Carbon atoms susceptible to a nucleophilic attack by bromide anion in pure RIL1_Br.

The results of thermal stability analysis of native PVA, pure RILs, and PVA-RIL membranes are depicted in Figure 5. The RIL1_Br and RIL2_Br thermal degradation profiles revealed four (Figure 5A) and three (Figure 5B) degradation stages, respectively. The first peak at ca. 100 °C, in the case of native RILs, is linked to the moisture release absorbed by highly hygroscopic native RILs [50]. The following stages between 100 and 300 °C can correspond to the nucleophilic substitution of bromide anion to the cation of an ionic liquid [51]. In the case of RIL1_Br, there are several sites susceptible to the nucleophilic attack (Figure 6). Bromide substitution to the methyl group (Figure 6, position A) or α-position of ester substituent (Figure 6, position B) in RIL1_Br results in the bromomethane or diethyl-2-bromomalonate formation [44].

Bromoethane and carbon dioxide can be also produced via the possible nucleophilic substitution of bromide anion to the ethyl group (Figure 6, position C) presented in ester substituent via the well-known Krapcho decarboxylation [52]. Ohtani et al. [51] pointed out the anion nucleophilic substitution and, thus, C–N bonds cleavage at the ethyl group due to the bromide anion presence in 1-ethyl-3-methylimidazolium bromide IL. Consequently, the formation of the following compounds: bromomethane and 1-ethylimidazole or bromoethane, ethylene and HBr was detected by pyrolysis-gas chromatograph [51].

The results of RIL2_Br TGA analysis indicate also the decomposition of ester groups (temperature of degradation between 140 and 250 °C) and imidazole ring (temperature of degradation between 250 and 320 °C) (Figure 5, B1 and B2). The initial decomposition temperature of RIL1_Br studied in this research is slightly lower comparing with the data obtained by Garcia et al. [53] for 3-methyl-1-hexyloxycarbonylmethylimidazolium bromide [C_6_EMeIm][Br] (T = 210 °C). The researchers pointed out the slight degradation temperature decrease (from 228 to 210 °C) as a result of the length reduction from 14 to 6 carbon atoms (from R = C_14_H_29_ to R = C_6_H_13_) of the alkyl group in ester-functionalized imidazolium-based ILs [53].

The increased degradation temperature at 10% weight loss up to 251 and 250 °C for PVA-9-RIL1_Br and PVA-9-RIL2_Br films, respectively, in respect to native PVA (243 °C) revealed the ability of RILs to raise thermal stability of PVA-based membranes (Table 2). The further raise of the RIL content, however, lowers the degradation temperature, which is more noticeable for PVA-RIL1_Br membranes (Table 2). Such tendency results from the inferior thermal stability of pure RIL1_Br comparing to pure RIL2_Br. RIL possesses more pronounced influence on the PVA-RILs membranes thermal stability at higher content of added RIL. Nevertheless, the elaborated PVA-RIL membranes are characterized by the thermal stability up to ca. 230 °C, which is suitable for the application in pervaporation and gas separation processes.

**Figure 7:**
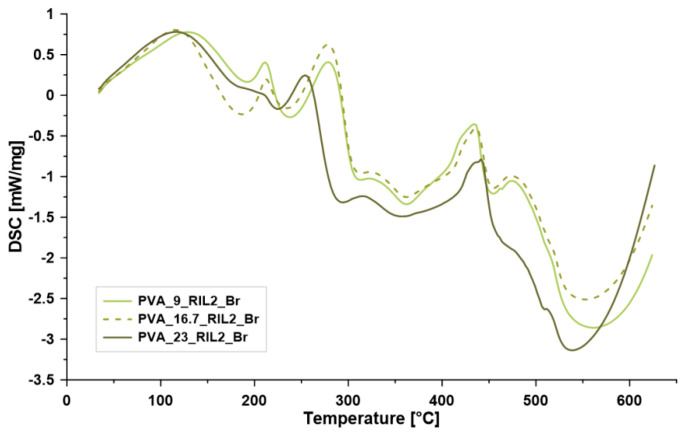
Differential scanning calorimetry (DSC) curves for the investigated membranes PVA-9-RIL2_Br, PVA-16.7-RIL2_Br, and PVA-23-RIL2_Br (exo-energetic processes—peaks down).

**Figure 8:**
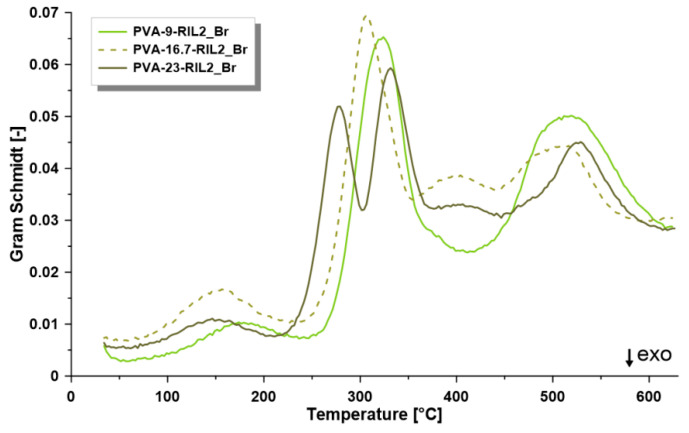
Gram–Schmidt diagram for PVA-9-RIL2_Br, PVA-16.7-RIL2_Br, and PVA-23-RIL2_Br membranes.

**Figure 9:**
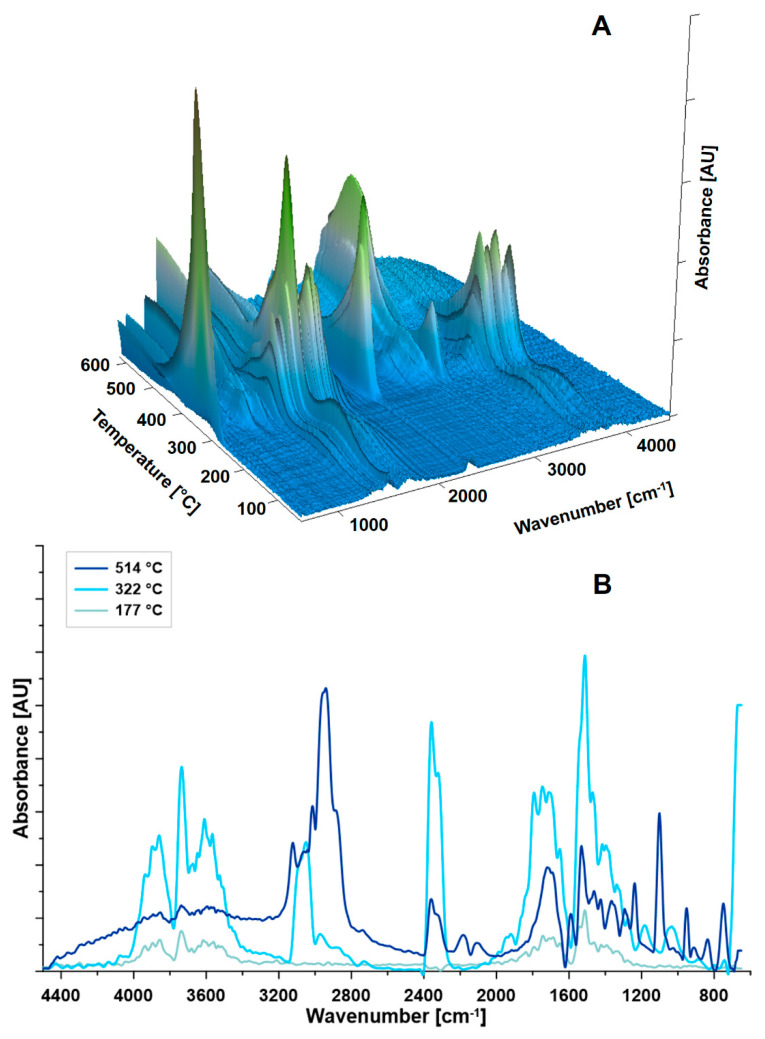
FTIR-TGA analysis of PVA-9-RIL2_Br: (**A**) 3D plot of IR spectra registered during the TGA analysis; (**B**) infrared (IR) spectra at selected temperatures.

Further and more detailed thermal analyses (differential scanning calorimetry (DSC) and TGA-FTIR) with additional online monitoring of gas products (Gram–Schmidt diagram) have been done by implementing TGA coupled with IR. The samples of PVA-based films with 9, 16.7, and 23 wt % of RIL2_Br were selected (Figure 7, Figure 8 and Figure 9). For the PVA membranes containing RIL2_Br, thermal features changed with the amount of the IL in the polymeric matrix. The samples with 9 and 16.7 RIL2_Br were characterized by similar thermal properties. On the other hand, the sample containing over 20 wt % of RIL (PVA-23-RIL2_Br) showed slightly different feature. For the two first membranes, the decomposition process took place practically at four steps (128, 288, 351, and 466 °C). Whereas, the decomposition process of PVA-23-RIL2_Br occurred at three steps at 126, 258, and 473 °C. Similarly to the RIL1_Br, the decomposition of samples containing RIL2_Br was related to the RIL decomposition (115–250 °C), loss of hydroxyl and acetate groups as a result of ester bonds breakage in PVA and RILs (200–300 °C), and, finally, to the PVA backbone degradation (400–500 °C) (Figure 5B1,B2).

The varied amount of RIL influenced the thermal properties as well as the energetic characterization of the membranes (Figure 7). The values of the reaction enthalpy were equal to 112.9 J g^−1^ (PVA-9-RIL2_Br), 126.4 J g^−1^ (PVA-16.7-RIL2_Br), and 83.6 J g^−1^ (PVA-23-RIL2_Br). Specific heat capacity of the samples was equal to 3.18 J g^−1^K^−1^ (PVA-9-RIL2_Br), 3.21 J g^−1^K^−1^ (PVA-16.7-RIL2_Br), and 3.93 J g^−1^K^−1^ (PVA-23-RIL2_Br). An increase of the specific heat capacity with the content of the RIL2_Br was related to the higher demand of the heat needed for the material decomposition. The different behavior of the PVA-23-RIL2_Br observed on the TGA, DTG, DSC as well as on the Gram–Schmidt diagram (Figure 8) corresponds to a very high concentration of ionic liquid possessing different properties from the polymeric matrix. The Gram–Schmidt diagram is a suitable tool for the more detailed TGA analysis showing at which temperature the gas product generation was observed during the thermal analysis. During the decomposition of ionic liquids (up to 200 °C) little amount of gas was produced. Then in the temperature range of 220–370 °C, the highest peaks were seen. The observation of two peaks (274 and 328 °C) in the mentioned range in the case of PVA-23-RIL2_Br sample was related to the highest level of the introduced ionic liquids. In the latter region, only the CO_2_ release has been found.

Basing on the Gram–Schmidt diagram, the temperature range (150–530 °C) was selected to monitor changes in IR spectra. Due to the increased amount of RIL in the investigated sample, the temperature onset moved to lower temperature. The detailed analyses for the investigated membranes are presented in Figure 9 and Figure S1 and was related to the monitoring of the gas product by registration its IR spectra. 3D IR spectra as the function of temperature gave inside view on the way of membranes decomposition. Additionally, the IR spectra were extracted and presented for the maxima of the temperature from Gram–Schmidt diagram. The most interesting behavior as a result of the higher level of the RIL in the membrane was noticed for PVA-23- RIL2_Br sample (Figure S1).

#### 3.1.3. Morphology and Surface Characterization

**Figure 10:**
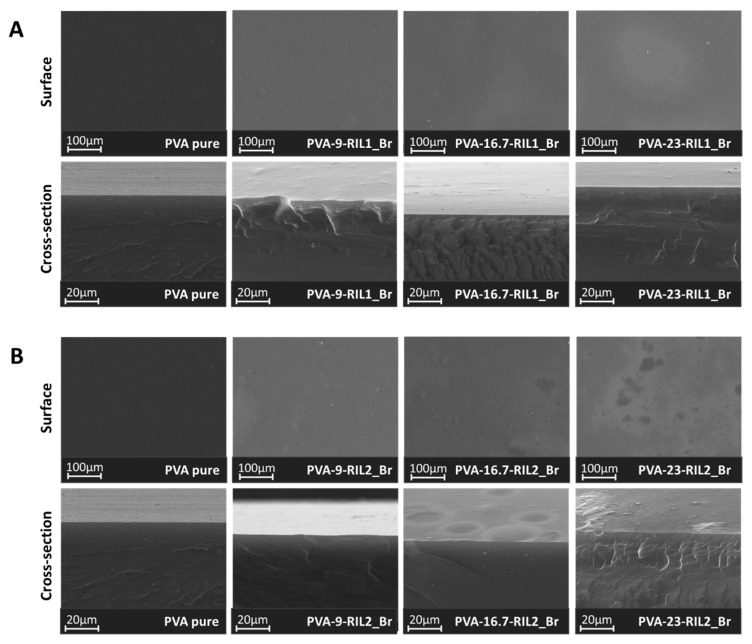
SEM images of (**A**) PVA-RIL1_Br and (**B**) PVA-RIL2_Br membranes surface and cross-section.

The cross-section images obtained in SEM analysis confirmed the nonporous and dense morphology of PVA-RIL1_Br and PVA-RIL2_Br films (Figure 10). The obtained films were heterogeneous, and the increasing content of RILs influenced the PVA-based membranes homogeneity, particularly in the case of PVA-RIL2_Br membranes. The increased heterogeneity of polymer membranes in presence of RIL1_Br and RIL2_Br was also observed in the case of the CAP-based membranes modified with RIL1_Br and RIL2_Br [36]. Kujawa et al. [36] indicated the decreased homogeneity of CAP-RILs structure and the presence of RIL-rich domains with the increasing RILs content. The observed slightly higher heterogeneity of PVA-RIL2_Br membranes may be correlated to the partial lower compatibility of PVA with RIL2_Br than to RIL1_Br.

The results of AFM analysis revealed that PVA-RIL membranes are characterized by the increased surface roughness with respect to pure PVA membrane whatever is the RILs type (Figure S2) which was reflected by the raised RIL-rich domains height and the higher *R_a_* and *R_q_* values (Table 3). The RILs content growth from 9 to 23 wt % smoothens the PVA-RILs membrane surface (Figure S2) what was demonstrated by diminishing *R_a_* values (Table 3). Such tendency was also indicated by Deng et al. [54] in the research on the cellulose acetate (CA)-based films modified with ether-functionalized pyridinium ILs ([E_n_Py][NTf_2_]). The researchers pointed out the CA films surface smoothening for an IL amount higher than 20 wt % as a result of liquid nature of tested [E_n_Py][NTf_2_] ionic liquid [54].

The increased surface roughness is a desirable feature of polymer membranes since it leads to the augmentation of the membrane active surface area, which is beneficial to the membrane performance in separation processes or fuel cell applications. Hooshyari et al. indicated the improved membrane proton conductivity due to the enhanced surface roughness of polybenzimidazole (PBI)-based membranes [55].

#### 3.1.4. Mechanical Performance

**Figure 11:**
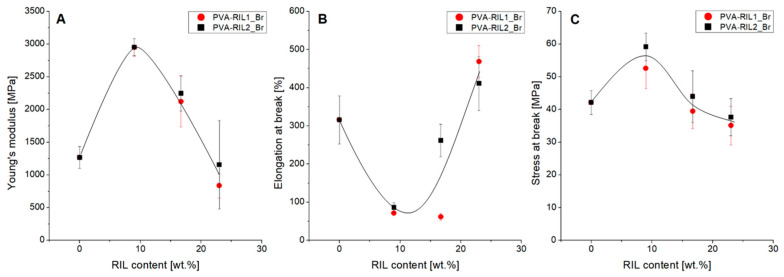
Mechanical properties of PVA-RIL films as a function of RIL content.

The results of tensile tests revealed no influence of RIL type on mechanical properties of PVA-RIL films but a significant influence of RIL presence on films ductility. The PVA-based films with low (9 wt %) RIL1_Br and RIL2_Br amount revealed the simultaneous rise of Young’s modulus (*YM*) value and reduction of elongation at break (*ε_max_*) value in respect to native PVA membrane (Figure 11). Such change resulted from the enhanced membrane stiffness induced by diminished PVA chains elasticity. RILs addition into the PVA-based matrix creates the RIL-PVA intramolecular interactions interfering with the polymer chains mobility and interactions between polymer chains. Furthermore, the use of low RIL amount enhances the hydrogen bond interactions between RILs imidazolium cation and PVA hydroxyl groups. The superior hydrogen bonds interactions play a role of a physical crosslinking between RILs and PVA molecules, thus increasing PVA-RIL films rigidity (Figure 11B).

The rise of RILs amount above 9 wt % results in *YM* reduction and *ε_max_* rise (Figure 11A,B, respectively). The higher RIL amount induces interactions between RIL molecules, which can lead to RIL-rich domains formation resulting in the diminished stiffness and improved flexibility in PVA-23-RIL1_Br and PVA-23-RIL2_Br membranes. It was noted that the high RILs amount (23 wt %) in the PVA-based membranes possesses the major impact on the membrane’s elasticity.

The outcome of PVA-based membranes modification with RILs demonstrated the dual RILs function: as a plasticizer and a physical junction promoter. The latter function was reflected by the initial *YM* and *σ_max_* increase and *ε_max_* decrease correlated with PVA-RIL membranes stiffening compared to pure PVA. The simultaneous shifts of the PVA vibrational bands (Figure 4) point out the predominant RIL impact on PVA chains mobility. Such phenomenon relates to the greater hydrogen bonding interactions between RILs cation and PVA hydroxyl groups inducing the RIL-PVA physical crosslinking. On the contrary, the high RILs amount addition resulted in the *YM* and *σ_max_* decrease and *ε_max_* increase, thus reflecting the reversed membranes mechanical properties, namely the improved PVA-RIL membranes elasticity. This observation was supported by SEM (Figure 10) and AFM (Table 3) analysis evidencing the desirable PVA-RIL compatibility that is characteristic for RILs playing a role of a plasticizer.

The mechanical properties observed for PVA-RIL membranes correspond well with the results of Yoon et al. [45] who investigated the PVA-based thermoplastic elastomer modified with 1-ethylpyridinium bromide IL [45]. The researchers indicated the shift of the hydroxyl stretching bands to higher frequencies as a function of pyridinium-based IL amount that resulted from the strong interaction between studied IL and PVA structure. The addition of 1-ethylpyridinium bromide IL led simultaneously to the *YM* drop from ca. 4000 MPa for pure PVA film to ca. 300 MPa for the PVA-based film with 70% of pyridinium-based IL [45].

### 3.2. Membrane Swelling Behavior in Water (H_2_O), Ethanol (EtOH), and Propan-2-ol (IPA)

#### 3.2.1. Degree of Swelling in Water (H_2_O), Ethanol (EtOH), and Propan-2-ol (IPA)

Membrane swelling measurements were conducted to investigate the sorption of H_2_O, EtOH and IPA into the PVA-RILs samples. The values of DS_e_ in water, ethanol and propan-2-ol sorption processes were shown in Table 4. The DS_e_ of all PVA-RILs membranes in water is distinctly higher than the DS_e_ in ethanol and propan-2-ol. The swelling degree results from the area change measurements showed that the PVA-ILs membranes exhibit weak swelling properties in ethanol and propan-2-ol. This phenomenon was also observed by Gimenes et al. [56] and Sajjan and Kariduraganavar [57]. It was found that pure ethanol was hardly dissolved in crosslinked PVA-based membranes and the swelling degree of gelatin incorporated PVA membranes in the mixture of 95 wt % of IPA and 5 wt % of water is even lower [56,57]. However, the swelling degree of PVA membranes in water is significantly higher. This is related to the strong interactions occurring between membrane and water molecules because of the interactive groups such as hydroxyl and –COO– ester groups present in PVA-RILs membranes which was confirmed by the FTIR-ATR results. Rynkowska et al. [30] investigated the swelling behavior of RIL1_Br incorporated CAP membranes. It was found that the incorporation of RIL1_Br enhanced the CAP-based membranes swelling in water comparing with pure CAP membrane. On the contrary, the CAP-based membranes swelling in organic solvents (EtOH and IPA) is reduced. This behavior results from the RIL1_Br hydrophilic character [30]. Those results are consistent with the data obtained in this research.

The DS_e_ of PVA-RILs membranes in water increased when the content of RILs increased (Table 4). The DS_e_ of PVA-RIL1_Br membrane in water increased from 137.7% to 348.9% when the content of RIL1_Br increased from 9 wt % to 33 wt %. The DS_e_ of PVA-RIL2_Br membrane in water increased from 95.7% to 512.3% when the content of RIL2_Br increased from 9 wt % to 23 wt %. When the content of RILs is low (9 wt %), the addition of RILs in PVA membranes reduced the chains flexibility and free volume availability due to the enhanced hydrogen bonding between hydroxyl groups of RIL and PVA which is confirmed by the FTIR-ATR spectra. The RILs act as physical crosslinker in PVA membranes. However, when the content of RILs is high (23 and 33 wt %), RILs-rich domains might be formed due to the interactions between RILs molecules. The crosslinking effect of RILs on PVA membranes reduced and the chains flexibility was enhanced, which can be confirmed by the results of mechanical analysis. Moreover, the –COO– ester group in PVA-RILs membrane increased when the content of RILs in PVA-RILs membranes is higher. Therefore, the DS_e_ of PVA-RILs membranes in water is significantly higher when increasing the RILs content in PVA-RILs membranes. Rynkowska et al. [58] investigated the physicochemical properties and pervaporation performance of CAP-based membranes with 1-methyl-3-(4-vinylbenzyl)-1H-imidazol-3-ium chloride polymerizable ionic liquid (PIL) and they found that the swelling degree of CAP-PIL membranes in H_2_O, EtOH and IPA raised with the PIL amount increase due to hydrophilic PIL character.

The sorption results of PVA-RIL membranes in H_2_O, EtOH and IPA showed that PVA-RIL membranes have significantly higher affinity to water and the results are consistent with the pervaporation findings of Kujawski et al. [59], Chaudhari et al. [60] and Rynkowska et al. [30] research. Kujawski et al. [59] investigated the properties of PVA-based membranes (Pervap^TM^) used for tetrafluoropropanol dewatering. It was found the process separation index of Pervap^TM^ 2200 and 2216 membranes approaches 5000 kg·m^−2^ h^−1^ and the water content in permeate of Pervap^TM^ 2200 and 2216 membranes is 99.8 wt % and 99.9 wt %, respectively when the feed containing 15 wt % of water was applied during pervaporation [59]. Chaudhari et al. investigated the properties of PVA-based membranes for propan-2-ol dehydration by pervaporation [60]. It was found that the content of water in permeate was as high as 99.4 wt % when the feed containing 20 wt % of water was applied during pervaporation. Rynkowska et al. [30] prepared RIL1_Br incorporated CAP membranes and investigated the efficiency of CAP-RIL1_Br membranes in pervaporation. The feed is the azeotropic mixture of H_2_O-IPA containing 12 wt % of water. It was found that CAP-RIL1_Br membranes are suitable for breaking H_2_O-IPA azeotrope. The water content in permeate for CAP-16.7-RIL1_Br membrane was found to be up to 98.1 wt %. The incorporation of RIL1_Br in CAP membrane results in a significant enhancement of separation factor β from 12 (pristine CAP membrane) to ca. 380 (CAP-16.7-RIL_Br membrane) [30].

Since an incorporation of RILs into PVA matrix, the state of ion pairs of ionic groups in PVA-RIL membranes is crucial to the swelling behavior of the membranes in different solvents [61,62,63]. Kujawski et al. [62] investigated the swelling properties of polyethylene/sulfonated poly(styrene-*co*-divinylbenzene) (PESS) ion exchange membranes in contact with solvents of different polarities. It was found that the membrane swelling decreased with the decreasing polarity of solvent. When the PESS membrane is equilibrated with water, the sulfonic anion-lithium counterion pairs are completely dissociated into hydrated ion-pairs. On the contrary, the ion-pairs are not dissociated when the membrane is equilibrated with alcohols such as ethanol and propan-2-ol. The swelling of ionic membranes are strongly influenced by the solvation and dissociation of ion-pairs [62]. Rynkowska et al. [63] investigated the swelling behavior of Nafion^®^ and IonClad™ membranes in polar and nonpolar solvents. It was found that both of the membranes showed a higher degree of swelling in polar solvents. When the ion exchange membranes are in contact with solvents of high polarity, the solvation shell is formed around the ion-pairs, subsequently, the ion-pairs are completely dissociated into separated ions resulting in the increase in the degree of swelling. These results are inconsistent with the data obtained in this research. The ion-pairs in PVA-RIL membranes are dissociated when the membranes are in contact with water. As a result, the DS_e_ of PVA-RIL membrane in contact with water is the highest (Table 4).

#### 3.2.2. Kinetics Analysis of Water Sorption Process

Since the PVA-RILs membranes have high affinity to water and the swelling degree in water is obviously high, the investigation on the kinetics of water sorption into PVA-RILs membranes would provide deeper understanding of the water sorption process. The water sorption kinetics for the PVA-RIL1_Br and PVA-RIL2_Br membranes was studied by fitting the experimental data by pseudo-first-order (PFO) and pseudo-second-order (PSO) models [64]. The nonlinear and linear forms of PFO and PSO models are gathered in Table 5.

**Figure 12:**
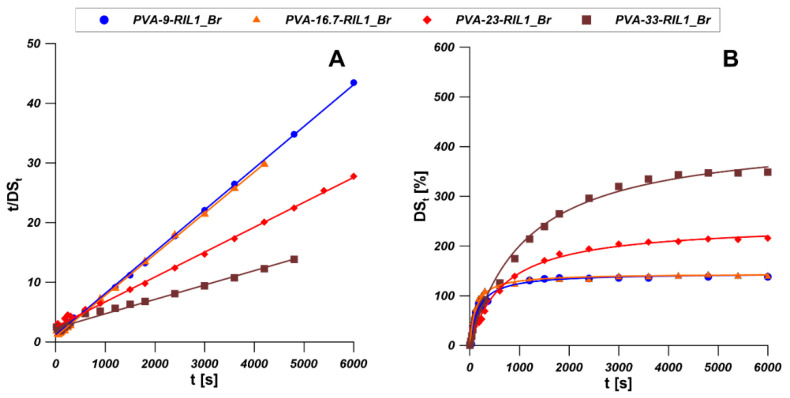
Experimental data of water sorption of PVA-RIL1_Br membranes fitted by linear (**A**) and non-linear (**B**) forms of PSO kinetic model.

As it is shown in Figure 12B, PVA-33-RIL1_Br membrane possesses the highest water sorption level which is more than 3 times of the water sorption level of PVA-9-RIL1_Br and PVA-16.7-RIL1_Br. The rise of RIL1_Br content in PVA membrane can enhance the water sorption level of the PVA membrane, however the water sorption level barely changed when the content of RIL1_Br in the PVA-based membrane raised from 9 wt % to 16.7 wt %. As it is shown in Figure 12A, membranes with a rather lower content of RIL1_Br possess relatively higher water sorption rate constants than the membranes with a higher content of RIL1_Br. PVA-33-RIL1_Br membrane possesses the lowest water sorption rate constant.

Sorption kinetics models can be employed to predict the equilibrium sorption capacity and elucidate the sorption mechanism. The experimental data were fitted by different models presented in Table 5 and Figure 12. The kinetic parameters for linear PFO and PSO models were acquired from the slope and the intercept of the plot of ln(DSe-DSt) vs time and t/DSt vs time respectively [65]. The corresponding kinetic parameters and correlation coefficients (R^2^) [65] are given Table 6. As shown in Table 6, the correlation coefficient R^2^ of PSO model is greater than that of PFO model. The experimental data were fitted the most accurately by the linear mode of PSO due to the high correlation coefficients (R^2^). Moreover, the estimated DS_e_ values of all PVA-RIL1_Br membranes correspond well with the experimental data. It was concluded that PSO model is more applicable for describing the sorption of water by PVA-RIL1_Br membranes.

**Figure 13:**
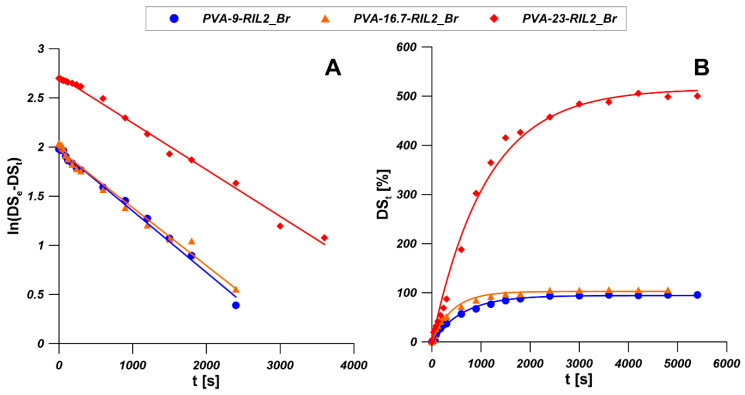
Experimental data of water sorption on PVA-RIL2_Br membranes fitted by linear (**A**) and non-linear (**B**) forms of PFO kinetic model.

Figure 13B presents the water sorption processes of PVA-RIL2_Br membranes. As it is shown, PVA-23-RIL2_Br membrane possesses the highest water sorption level which is near five times of PVA-9-RIL2_Br and PVA-16.7-RIL2_Br membranes. Increasing content of RIL2_Br in PVA membrane can increase the water sorption level, however, the water sorption level changed slightly when the concentration of RIL2_Br in the PVA membrane is lower than 16.7 wt %. The experimental data were fitted by the models shown in Table 5 and illustrated in Figure 13A. The corresponding kinetic parameters and correlation coefficients (R^2^) [65] are given in Table 6. It was found that for all PVA-RIL2_Br membranes, the PFO kinetics model was more applicable for the description of the water sorption process as demonstrated by the higher correlation coefficients (R^2^) compared to the PSO kinetics model. Moreover, the DS_e_ values estimated by PFO kinetics model are in a better agreement with experimental data.

In comparison with these two types of PVA membranes, the increasing content of RIL1_Br and RIL2_Br shows a similar effect on the water sorption level of PVA membranes. However, the equilibrium water sorption capacity and the water sorption rate constant of PVA-RIL1_Br and PVA-RIL2_Br membranes were estimated by PSO kinetics model and PFO kinetics model, respectively.

## 4. Conclusions

In the presented work, PVA based membranes incorporated with ester-functionalized imidazolium-based reactive ionic liquids (RILs) were successfully elaborated by dry phase inversion method. The effects of RILs type and the content of RILs on the physicochemical properties of prepared membranes were evaluated. It was found that the thermal and mechanical stability of PVA membranes was enhanced after the incorporation of RILs due to the hydrogen bonding between PVA chains and RILs. However, the stability was reduced with the level of the RIL in the polymeric matrix. The thermal behavior of the polymeric films was investigated by FTIR-TGA analysis presenting complexity and instability of the material with 23 wt % of the RIL. Membranes containing 9 wt % of RILs exhibited the best thermal stability and mechanical strength. Thus, 9 wt % is the optimal amount for the fabrication of PVA-RILs membranes. The RILs-rich domains were observed by using the AFM and SEM analyses for membranes contain higher content of RILs. Membrane swelling behavior in water (H_2_O), ethanol (EtOH), and propan-2-ol (IPA) and the kinetics of water sorption process were investigated. It was found that PVA-RILs membranes possess the highest affinity to water and the equilibrium water sorption capacity and the water sorption rate constant of PVA-RIL1_Br and PVA-RIL2_Br membranes were estimated by PSO kinetics model and PFO kinetics model, respectively. It is concluded that the type of RILs and the amount of the RILs in the polymer matrix have significant influence on the membrane swelling behavior and the kinetics of water sorption.

## Figures and Tables

**Table 1:** Absorption Bands Assignments in RIL1_Br [30] and RIL2_Br.

Absorption Bands	Wavenumber [cm^−1^]	
	RIL1_Br [30]	RIL2_Br
Water presence in a sample	-	3425
C–H stretching (imidazolium ring)	3070	3082
C–H stretching (alkyl)	2993	2982
C=O stretching (–COO– ester group)	1759	1745
Imidazolium ring stretching	1556	1572
C–O stretching (–COO– ester group)	1221, 1263	1225
C–H scissoring deformation (CH_2_ in methylene group)	1471	1470
C–H asymmetric deformation (CH_3_ group)	1444	1439
H–C–C and H–C–N bending (imidazolium ring)	1182	1174
C–H in-plane deformation (imidazolium ring)	839	876
C–H out-of-plane deformation (imidazolium ring)	750	766

**Table 2:** Degradation Temperatures of PVA-RIL1_Br and PVA-RIL2_Br Films.

Membranes	Degradation Temperature [°C]	
	at 5% Weight Loss	at 10% Weight Loss
PVA	163 ± 2	243 ± 2
RIL1_Br	97 ± 2	118 ± 2
PVA-9-RIL1_Br	183 ± 2	251 ± 2
PVA-16.7-RIL1_Br	126 ± 2	234 ± 2
PVA-23-RIL1_Br	120 ± 2	235 ± 2
RIL2_Br	90 ± 2	161 ± 2
PVA-9-RIL2_Br	148 ± 2	250 ± 2
PVA-16.7-RIL2_Br	122 ± 2	242 ± 2
PVA-23-RIL2_Br	126 ± 2	228 ± 2

**Table 3:** Roughness Parameters (*R*_q_ and *R*_a_) of pristine PVA and PVA-based membranes with RIL1_Br and RIL2_Br.

Parameters	*R_q_* [nm]	*R_a_* [nm]
Scanning Area	5 μm × 5 μm	
PVA	3.2 ± 0.8	2.3 ± 0.5
PVA-9-RIL1_Br	14.0 ± 1.3	10.2 ± 1.1
PVA-16.7-RIL1_Br	7.5 ± 2.7	5.6 ± 2.6
PVA-23-RIL1_Br	4.4 ± 2.5	2.7 ± 1.7
PVA-9-RIL2_Br	7.9 ± 3.4	6.5 ± 2.8
PVA-16.7-RIL2_Br	6.8 ± 3.2	5.1 ± 2.4
PVA-23-RIL2_Br	6.3 ± 1.1	5.1 ± 0.8

**Table 4:** The DS_e_ Values of PVA-RIL1 and PVA-RIL2 Membranes in Water, Ethanol and Propan-2-ol.

Membranes	DS_e_ (%)		
	Water	Ethanol	Propan-2-ol
Pristine PVA	98.6	3.2	4.7
PVA-9-RIL1_Br	137.7	0.8	2.0
PVA-16.7-RIL1_Br	141.0	2.9	1.9
PVA-23-RIL1_Br	216.4	2.8	2.2
PVA-33-RIL1_Br	348.9	2.9	2.6
PVA-9-RIL2_Br	95.7	1.2	0.6
PVA-16.7-RIL2_Br	108.5	0.8	1.2
PVA-23-RIL2_Br	512.3	0.7	2.7

**Table 5:** Differential and Linear Forms of Pseudo-First-Order (PFO) and Pseudo-Second-Order (PSO) Models [65].

Kinetics Models	Non-Linear Equation		Linear Equation	
PFO	${\mathrm{DS}}_{t}{\;=\;\mathrm{DS}}_{e}(1-e^{{-k}_{1}t}$)	(3)	$\ln\left({\mathrm{DS}}_{e}-{\mathrm{DS}}_{t}\right){=\mathrm{lnDS}}_{e}-k_{1}t$	(4)
PSO	${\mathrm{DS}}_{t}=\frac{k_{2}{\mathrm{DS}}_{e}^{2}t}{{1+k}_{2}{\mathrm{DS}}_{e}t}$	(5)	$\frac{t}{{\mathrm{DS}}_{t}}=\frac{1}{k_{2}{\mathrm{DS}}_{e}^{2}}+\frac{t}{{\mathrm{DS}}_{e}}$	(6)

k_1_ (s^−1^) is the PFO rate constant, k_2_ (s^−1^) is the PSO rate constant, DS_t_ (%) and DS_e_ (%) are the swelling degree of membranes at time t (s) and equilibrium, respectively.

**Table 6:** The Estimated Kinetic Parameters of Two Sorption Models for Water Sorption of PVA-RIL1_Br and PVA-RIL2-Br Membranes at Room Temperature.

PVA-RIL1_Br	Parameters	RIL1_Br (wt %)				RIL2_Br (wt %)		
		9	16.7	23	33	9	16.7	23
Experiment	DS_e_ (%)	138	141	216	349	96	109	512
FO (non-linear mode)	DS_e_ (%)	135	136	210	350	94	103	515
	k_1_ (s^−1^)	4.22 × 10^−3^	6.21 × 10^−3^	1.26 × 10^−3^	8.27 × 10^−4^	1.58 × 10^−3^	2.34 × 10^−3^	9.07 × 10^−4^
	R^2^	0.979	0.987	0.992	0.991	0.994	0.988	0.993
PFO (linear mode)	DS_e_ (%)	58	46	184	378	94	94	524
	k_1_ (s^−1^)	6.91 × 10^−4^	9.21 × 10^−4^	9.21 × 10^−4^	9.21 × 10^−4^	1.44 × 10^−3^	1.36 × 10^−3^	1.10 × 10^−3^
	R^2^	0.716	0.546	0986	0.982	0.994	0.983	0.994
PSO (non-linear mode)	DS_e_ (%)	146	145	245	423	109	117	652
	k_2_ (s^−1^)	4.00 × 10^−5^	5.95 × 10^−5^	5.97 × 10^−6^	2.09 × 10^−6^	1.71 × 10^−5^	2.47 × 10^−5^	1.32 × 10^−6^
	R^2^	0.980	0.993	0.995	0.994	0.994	0.995	0.983
PSO (linear mode)	DS_e_ (%)	142	145	238	416	128	125	714
	k_2_ (s^−1^)	4.37 × 10^−5^	5.15 × 10^−5^	6.96 × 10^−6^	2.51 × 10^−6^	8.10 × 10^−6^	1.63 × 10^−6^	8.11 × 10^−7^
	R^2^	0.999	0.999	0.997	0.987	0.879	0.972	0.944

## Supplementary Materials

The supplementary materials are available online at https://www.mdpi.com/2073-4360/12/9/1958/s1, Figure S1: PVA-16.7-RIL2_Br-A1–3D plot of IR spectra register during the TGA analysis, A2–IR spectra at selected temperatures; PVA-23-RIL2_Br-B1–3D plot of IR spectra register during the TGA analysis, B2–IR spectra at selected temperatures, Figure S2: 3D surface profile of pristine PVA and PVA-based membranes blended with RIL1_Br and RIL2_Br at different concentrations.
