# Supplementary material for: The Impact of Reactive Ionic Liquids Addition on the Physicochemical and Sorption Properties of Poly(Vinyl Alcohol)-Based Films
Source: Polymers (Basel). 2020 Aug 29;12(9):1958. doi: 10.3390/polym12091958 (PMC7565177; doi:10.3390/polym12091958)
Supplement: Supplementary file 1 [file polymers-12-01958-s001.pdf]

Supporting information

The impact of reactive ionic liquids addition on the physicochemical and sorption properties of poly(vinyl alcohol)-based films

Guoqiang Li, Edyta Rynkowska, Kateryna Fatyeyeva, Joanna Kujawa, Krzysztof Dzieszkowski, Andrzej Wolan, Stephane Marais, Corinne Chappey, Zbigniew Rafiński, and Wojciech Kujawski

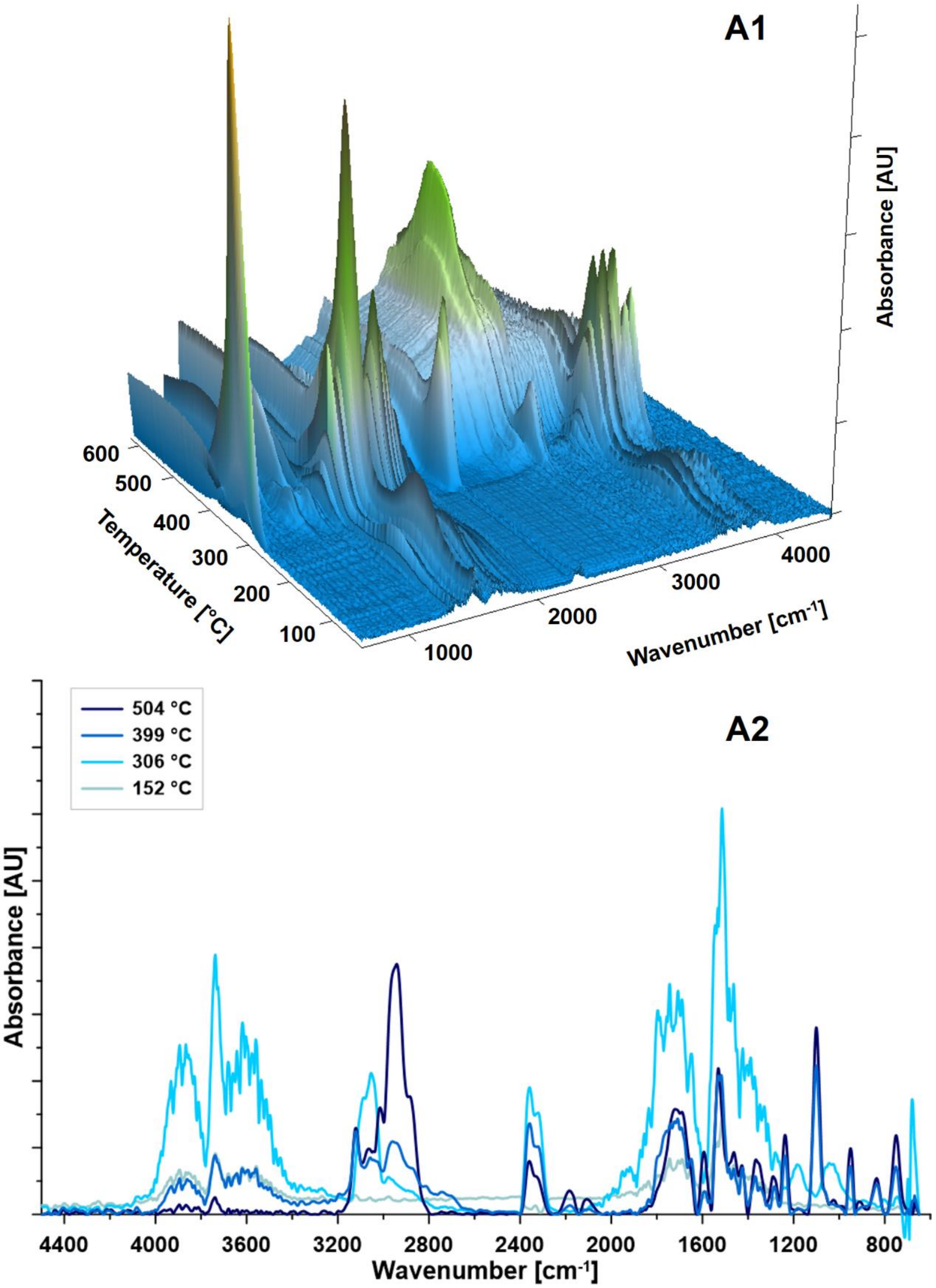
A1
Absorbance [AU]
600
500
400
300
200
100
Temperature [°C]
1000
2000
3000
4000
Wavenumber [cm-1]
A2
504 °C
399 °C
306 °C
152 °C
Absorbance [AU]
4400
4000
3600
3200
2800
2400
2000
1600
1200
800
Wavenumber [cm-1]

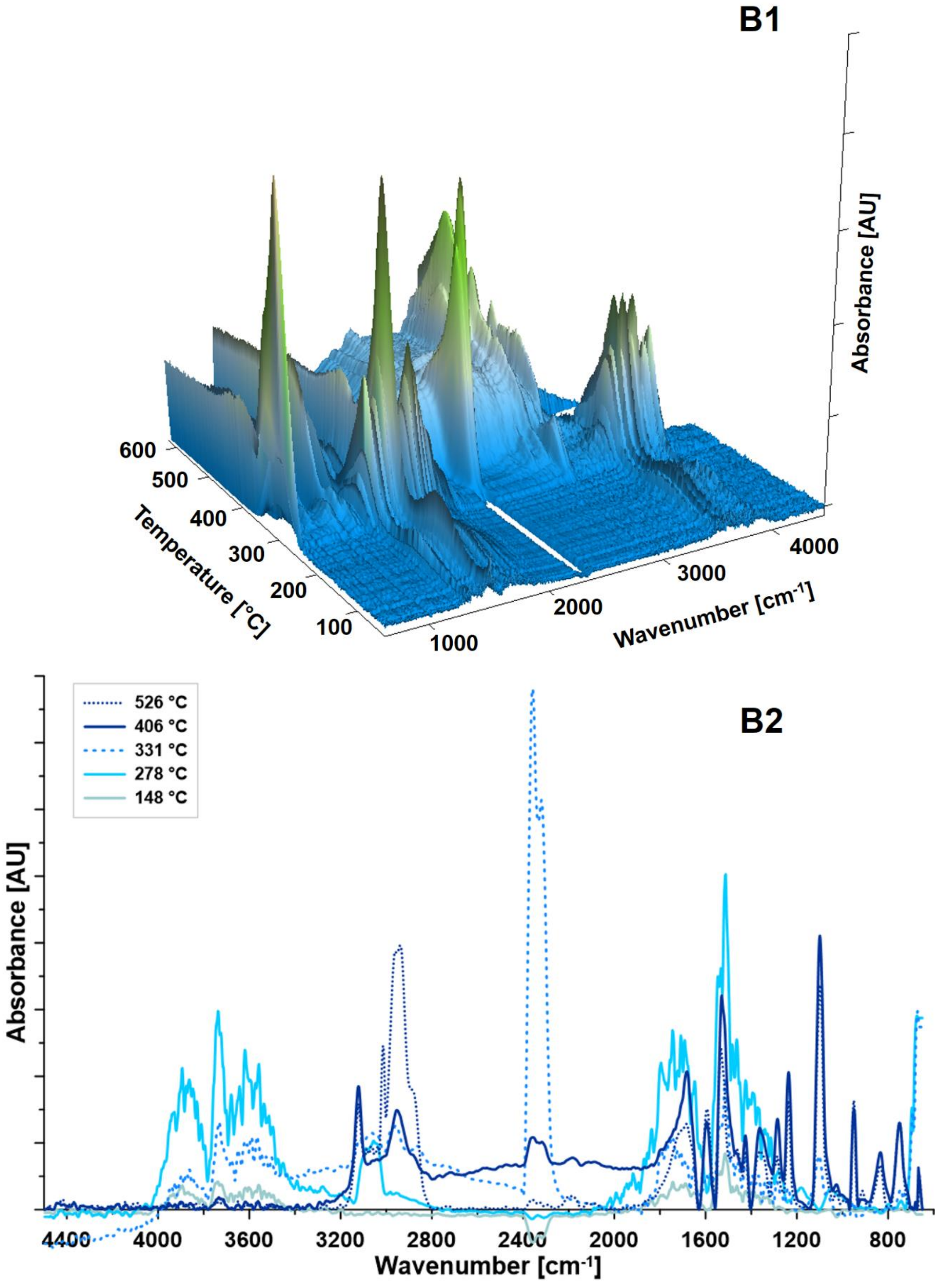

Figure S1. PVA-16.7-RIL2_Br - A1 – 3D plot of IR spectra register during the TGA analysis, A2 – IR spectra at selected temperatures; PVA-23-RIL2_Br - B1 – 3D plot of IR spectra register during the TGA analysis, B2 – IR spectra at selected temperatures.

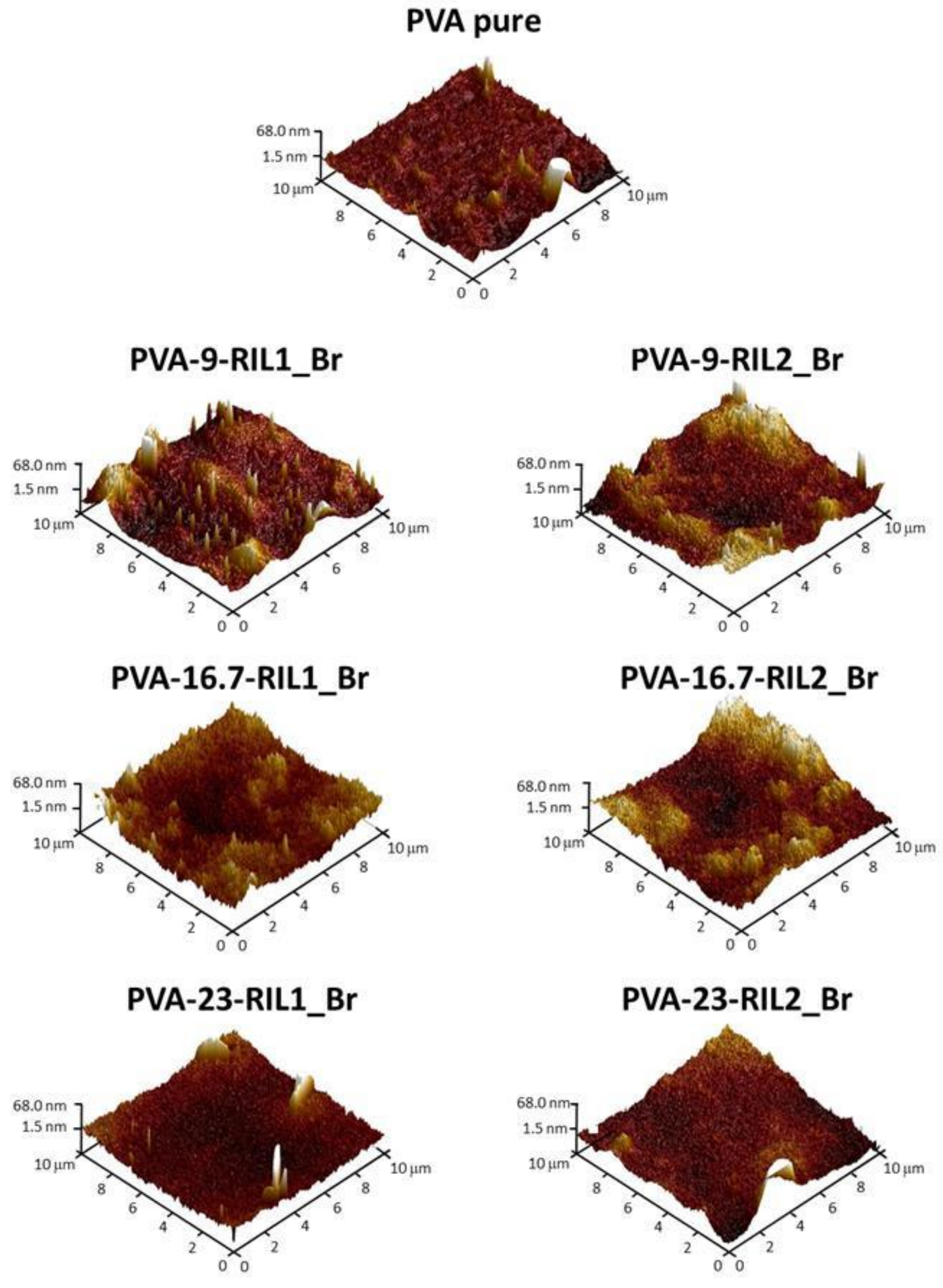

Figure S2. 3D surface profile of pristine PVA and PVA-based membranes blended with RIL1_Br and RIL2_Br at different concentrations.
